# Nomogram based on a circular RNA biomarker for predicting the likelihood of successful sperm retrieval *via* microdissection testicular sperm extraction in patients with idiopathic non-obstructive azoospermia

**DOI:** 10.3389/fendo.2022.1109807

**Published:** 2023-01-17

**Authors:** Shengjia Shi, Tianwei Wang, Lei Wang, Mingjuan Wang

**Affiliations:** ^1^ Reproductive Center, Northwest Women’s and Children’s Hospital, Xi’an, China; ^2^ Department of Pathology, Northwest Women’s and Children’s Hospital, Xi’an, China

**Keywords:** circ_MGLL, idiopathic non-obstructive azoospermia, microdissection testicular sperm extraction, sperm retrieval, decision curve analysis

## Abstract

**Background:**

Many circular RNAs (circRNAs) are specifically expressed in the testes and seminal plasma of patients with non-obstructive azoospermia (NOA), highlighting them as potential predictors of microdissection testicular sperm extraction (micro-TESE) outcomes. Although research has indicated that circular RNA monoglyceride lipase (circ_MGLL) is highly expressed in the testicular tissues of patients with NOA, the association between circ_MGLL expression and sperm retrieval outcomes (SROs) in patients with idiopathic non-obstructive azoospermia (iNOA) receiving micro-TESE remains unclear.

**Methods:**

This single-center, retrospective cohort study enrolled 114 patients with iNOA who underwent micro-TESE at Northwest Women’s and Children’s Hospital from January 2017 to November 2021. A logistic regression model was used to examine associations between SRO and circ_MGLL expression in testicular tissues, the results of which were used in conjunction with previous findings to establish a nomogram. The predictive performance of the circ_MGLL-based nomogram was evaluated *via* calibration curves, receiver operating characteristic curves, and decision curve analysis (DCA) using an internal validation method.

**Results:**

The generalized additive model indicated that the probability of successful SRO for micro-TESE decreased as circ_MGLL expression increased in testicular tissues. Across the entire cohort, univariate logistic regression analysis revealed that circ_MGLL expression was inversely associated with SRO in patients with NOA. This trend did not change after stratification according to age, body mass index, testicular volume, follicle-stimulating hormone (FSH) level, luteinizing hormone (LH) level, testosterone (T) level, or pathological type (or after adjusting for these confounders) (odds ratio <1, *P* < 0.001). A nomogram was then generated by integrating circ_MGLL, pathological types, and FSH, LH, and T levels. The circ_MGLL-based predictive model achieved satisfactory discrimination, with an area under the curve of 0.857, and the calibration curves demonstrated impressive agreement. The DCA indicated that the net clinical benefit of the circ_MGLL-based predictive model was greater than that of circ_MGLL alone.

**Conclusion:**

circ_MGLL is significantly associated with the SRO of micro-TESE in patients with iNOA. The circ_MGLL-based nomogram developed in the current study can predict successful SRO with high accuracy.

## 1 Introduction

Azoospermia is characterized by the complete absence of spermatozoa in the ejaculate semen, occurring in approximately 1% of all men and 10%–15% of infertile men (1). However, in some cases, intracytoplasmic sperm injection treatment can be performed after obtaining sperm through microdissection testicular sperm extraction (micro-TESE) (2). Among the types of non-obstructive azoospermia (NOA), idiopathic NOA (iNOA) presents a particular clinical challenge given the lack of a clear etiology, absence of effective treatments, and poorest sperm retrieval outcomes (SROs) following micro-TESE (3, 4). Regrettably, micro-TESE is only successful in approximately 50% of patients with NOA (5, 6). Although research has identified various factors related to SRO, such as testicular volume (TV) and hormonal levels [*e*.*g*., follicle-stimulating hormone (FSH), luteinizing hormone (LH), and testosterone (T)] (5), reliable molecular biomarkers for predicting SROs following micro-TESE in patients with iNOA remain to be identified.

Testicular biopsy is an important invasive procedure for evaluating testicular spermatogenesis (7). However, whether testicular biopsy is necessary before micro-TESE remains controversial. Because spermatogenic foci are few and scattered in patients with iNOA, biopsied testicular tissues may not reflect spermatogenesis in other parts of the testis. Thus, some studies have pointed out that testicular biopsy only contributes to the pathological classification of the biopsied tissues and has limited value in the prediction of SROs following micro-TESE (8). Meanwhile, open testicular biopsy increases the risk of testicular injury and bleeding (9). However, other studies have confirmed that testicular biopsy is of major predictive significance in the context of micro-TESE outcomes. Mao et al. reported extremely poor SROs (11% success) in patients with iNOA whose biopsy results indicated an absence of sperm in both the intraoperative microscopic examination and the post-pathological examination—a rate far below that observed for patients with iNOA exhibiting identifiable sperm in even just one of the two examinations (10). Cao et al. also found significantly different profiles of PIWI-interacting RNA (piRNA) between successful and failed micro-TESE procedures among patients with NOA, providing insight into strategies for identifying biomarkers that can predict residual spermatogenic loci in this population (11). Our collaborators also reported obvious upregulation of beclin-1 in the Sertoli cell-only syndrome (SCOS) group, which was further identified as an important predictor of SRO failure in micro-TESE (12). Taken together, these findings indicate that different histopathological outcomes and differentially expressed genes in testicular tissues obtained during micro-TESE or testicular biopsy may, in part, reflect the extent of spermatogenesis within the whole testis, suggestive of the potential value for predicting SROs following micro-TESE in patients with NOA.

Circular RNAs (circRNAs) are a new class of endogenous non-coding RNAs that covalently bind to a closed circular structure without a 5′ end cap and a 3′ end poly(A) tail (13). Several studies have confirmed that circRNAs play a pivotal role in regulating gene expression and cell fate (14). Many circular RNAs (circRNAs) are specifically expressed in the testis and seminal plasma of patients with NOA (15) and can be used as predictors of the outcome of micro-TESE (16). However, research regarding the expression and function of circRNAs in spermatogenesis is still in its infancy. Our collaborators have shown that approximately 1,000 circRNAs, including circ_0008045 (circular RNA monoglyceride lipase, circ_MGLL), are differentially dysregulated in testicular tissues between NOA and obstructive azoospermia (17). Previous studies have indicated that the expression of monoglyceride lipase (MGLL), an important downstream factor involved in triglyceride catabolism, is very low in germ cells but is specifically abundant in Sertoli cells, indicating that MGLL is a potential functional regulator of Sertoli cells (18). More importantly, silencing the expression of circ_MGLL results in an increase in the proliferation of Sertoli cells (19). However, many studies have reported that changes in the population or function of Sertoli cells are among the main factors leading to defective spermatogenesis (20). Nevertheless, the clinical relevance of circ_MGLL in patients with iNOA remains to be determined.

In the current study, we discovered that circ_MGLL, a circRNA generated from the circularization of the MGLL gene, was significantly upregulated in the testicular tissues of patients with iNOA in the SRO-failure group. Detailed univariate and multivariate logistic analyses were conducted to further clarify the relationship between circ_MGLL and SRO. Furthermore, stratified analyses were performed to determine whether the correlation between circ_MGLL and SRO remained within different subgroups, thus distinguishing our study from previous research. Finally, receiver operating characteristic (ROC) curve and decision curve analysis (DCA) were performed to analyze the ability of circ_MGLL to predict SROs.

## 2 Materials and methods

### 2.1 Patients and specimens

This single-center, retrospective cohort study enrolled 114 patients with iNOA who underwent micro-TESE at Northwest Women’s and Children’s Hospital from January 2017 to November 2021. The inclusion criteria for all cases have been described in our previously published article (12). The study was approved by the Ethics Review Board of Northwest Women’s and Children’s Hospital (ethical review number: 2021-XBFE-023). Written informed consent for the use of their testis biopsies and anonymized data for research purposes was obtained from the participants before micro-TESE. All procedures were performed in accordance with the ethical standards of the responsible committee on human experimentation or the Helsinki Declaration of 1975.

The inclusion criteria were as follows (1): a history of infertility of not less than 1 year, (2) no sperm detected in three semen analyses, and (3) completion of micro-TESE to obtain sperm. The exclusion criteria were (1) a diagnosis of congenital absence of the vas deferens, (2) abnormal seminal fructose or neutral α-glucosidase, (3) absence of seminal glands, (4) other obstructive factors accounting for azoospermia, (5) lack of Johnsen scores after micro-TESE surgery, (6) abnormal chromosome karyotype, (7) microdeletions in the azoospermia factor (AZF) c region of the Y chromosome, (8) a history of cryptorchidism, and (9) a history of cryptorchidism.

### 2.2 micro-TESE

The patients underwent the micro-TESE procedure as described in our previous study (12). Briefly, the TV was measured in a standard surgical room. Then, a longitudinal incision on the tunica albuginea of the testes was made to reveal the seminiferous tubules. Tubules with a full appearance and opacity, suggestive of possible sperm production, were gently dissected and placed in a Petri dish. An experienced embryologist dissected the seminiferous tubules and assessed the presence of sperms using a specialized microscope. A positive result was confirmed when at least one sperm sample was obtained. Thereafter, a large fragment of testicular tissue measuring approximately 8 × 4 × 3 mm^3^ was cut for histopathological examination, regardless of whether the sperm had been harvested successfully.

### 2.3 Pathological examination and Johnsen score

Testicular tissue samples were fixed in Bouin’s solution for 24 h and embedded in paraffin. The slides were then stained with hematoxylin and eosin solution. Subsequently, all slides were observed under a microscope by two pathologists. The level of spermatogenesis in each testicular biopsy sample was measured based on the Johnsen score system as previously described (21). Patients with iNOA who had Johnsen scores of 1 to 2 were identified as having Sertoli cell-only syndrome (SCOS), those with Johnsen scores of 3–7 were identified as having maturation arrest (MA), and those with Johnsen scores of 8 to 9 were identified as having hypo-spermatogenesis (HS). Typical photomicrographs of the different Johnsen scores are presented in **Supplementary Figure S1**.

### 2.4 RNA extraction and quantitative real-time polymerase chain reaction

Total RNA was extracted from testicular tissues using a paraffin-embedded tissue section total RNA extraction kit (Tiangen, Beijing, China) in accordance with the manufacturer’s instructions. For circ_MGLL quantification, a one-step prime-script-circRNA cDNA synthesis kit was used to convert the total RNA to cDNA. Then, the SYBR premix Ex Taq kit (Takara, Japan) and specific circRNA LNATM PCR primers (Exiqon, Denmark) were used to perform real-time polymerase chain reaction (RT-PCR). Data were analyzed using 7500 software v.2.0.1 (Applied Biosystems, USA), with the automatic Ct setting for adapting the baseline and threshold for Ct determination. Each sample was examined in triplicate, and the amount of PCR product produced was normalized to GAPDH. The primers used for circ_MGLL were 5′-GCCTACCTGCTCATGGAGTT-3′ (forward) and 5′-AGACGGCATTCAGCAGTTG-3′ (reverse). The primers used for GAPDH were 5′-AATCCCATCACCATCTTCCA-3′ (forward) and 5′-TGGACTCCACGACGTACTCA-3′ (reverse).

### 2.5 Statistical analyses

Statistical analysis was performed using IBM SPSS version 23.0 (International Business Machines Corporation, USA), R software (http://www.R-project.org, The R Foundation), and EmpowerStats (http://www.empowerstats.com, X&Y Solutions, Inc., Boston, MA, USA). Continuous variables are presented as the mean ± standard deviation (SD) and were compared using one-way analysis of variance (ANOVA) and the Kruskal–Wallis test in cases of normally and non-normally distributed data, respectively. Categorical data are presented as percentages and were compared using chi-square tests. A generalized additive model was used for smooth curve fitting to evaluate the relationship between circ_MGLL expression and successful SRO probability. The odds ratios (OR) and 95% confidence intervals (CIs) of SRO according to adjusted or unadjusted for potential confounders were evaluated using univariate and multivariate logistic regression models. Stratified analyses were conducted according to age (years), body mass index (BMI, kg/m^2^), average TV (ml), FSH (IU/L), LH (IU/L), T (ng/dl), and pathological type. A nomogram integrating circ_MGLL, pathological type, and serum hormone levels was established to predict the probability of successful SRO. Subsequently, the original data were divided into a training set (*n* = 58, successful SRO: 29, failed SRO: 29) and a validation set (*n* = 56, successful SRO: 18, failed SRO: 38), and the predictive performance of the circ_MGLL-based nomogram was evaluated in the training set and internally validated *via* bootstrapping with 500 resamples. The accuracy of the circ_MGLL-based nomogram was assessed using calibration curves, ROC, and DCA. All *P*-values were generated using two-sided tests, and statistical significance was set at *P <*0.05.

## 3 Results

### 3.1 Participants’ characteristics

**Figure 1:**
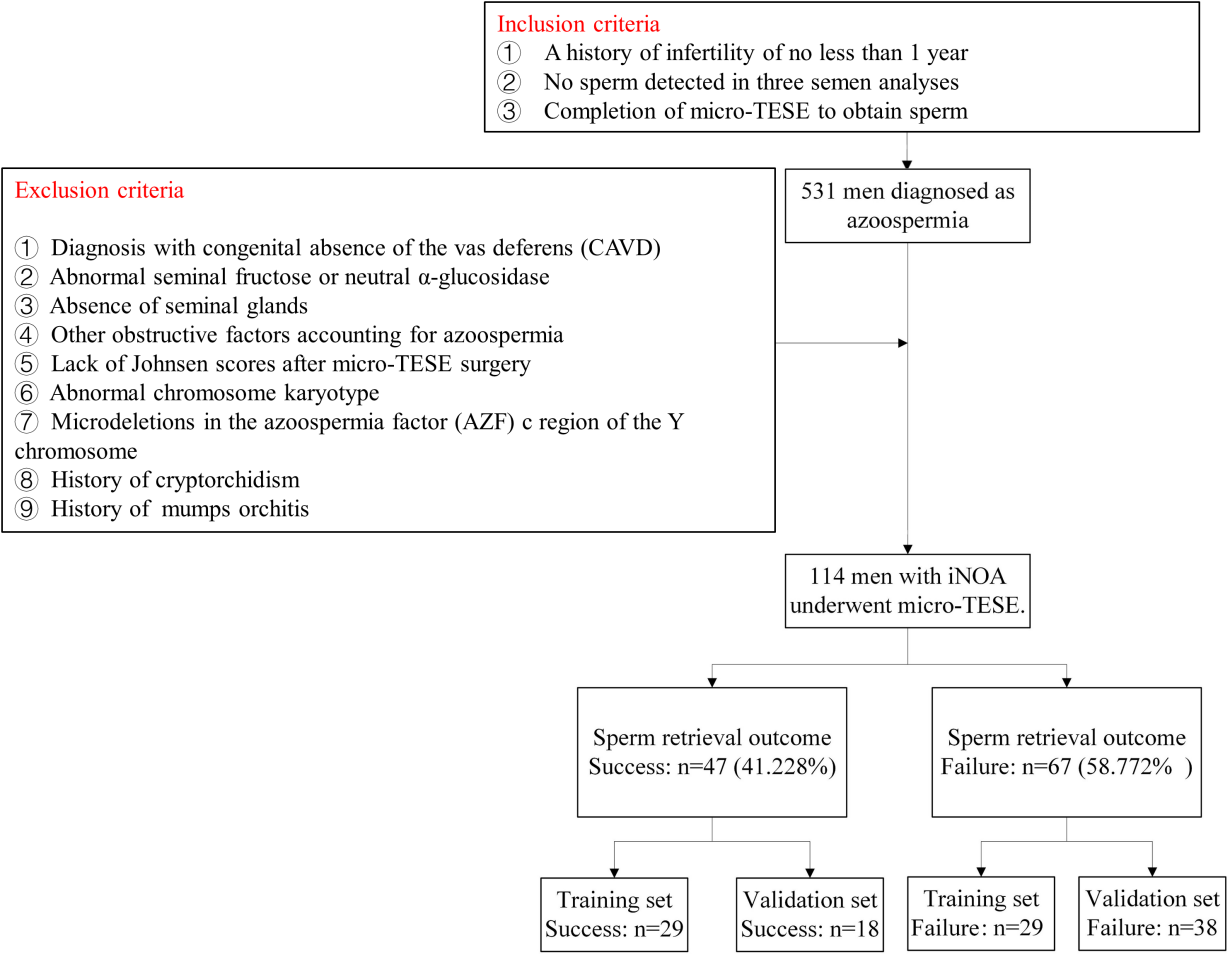
Flow diagram of patient selection.

In accordance with the inclusion and exclusion criteria depicted in **Figure 1**, 114 patients with iNOA in our hospital were included in the final analysis. **Table 1** shows the clinical characteristics of these 114 patients with iNOA who were classified into success (47, 41.228%) and failure (67, 58.772%) groups based on the SRO of micro-TESE. There were no significant differences in age, TV, FSH levels, LH levels, or T levels between the two groups (**Table 1**). Although successful SROs were more common in patients with pathological type MA or HS than in those with type SCOS, the differences among the three groups were not significant (*P* = 0.067, **Table 1**).

**Table 1:** Clinical characteristics of idiopathic non-obstructive azoospermia patients receiving micro-testicular sperm extraction.

Variables	Failure (*n* = 67)	Success (*n* = 47)	*P*
C**ircular RNA monoglyceride lipase**	6.617 ± 2.124	3.903 ± 1.698	<0.001
**Age (years)**	30.090 ± 3.311	30.064 ± 3.300	0.967
**B**MI (kg* m^-2^)	24.784 ± 3.117	25.564 ± 3.803	0.232
F**ollicle-stimulating hormone (IU/L)**	22.033 ± 13.166	21.413 ± 10.930	0.791
L**uteinizing hormone (IU/L)**	9.944 ± 8.951	8.980 ± 4.865	0.504
**Testosterone (ng/**dl**)**	412.914 ± 191.582	392.904 ± 178.437	0.574
**TV (ml)**			0.282
**≤10** ml	61.2 (41)	51.1 (24)	
**>10** ml	38.8 (26)	48.9 (23)	
**Pathological type**			0.067
**SCOS**	49.3 (33)	34.0 (16)	
**MA**	47.8 (32)	53.2 (25)	
**HS**	3.0 (2)	12.8 (6)	

BMI, body mass index; TV, testicular volume; SCOS, Sertoli cell-only syndrome; MA, maturation arrest; HS, hypo-spermatogenesis.

### 3.2 Relative expression of circ_MGLL in the testicular tissues of patients with iNOA

**Figure 2:**
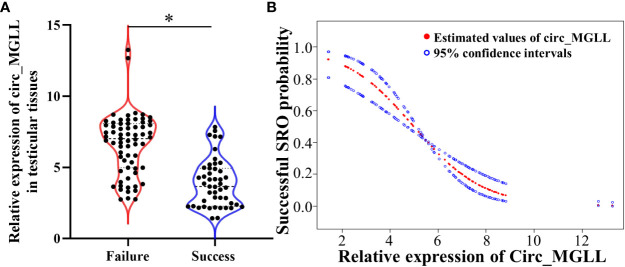
Relationships between circ_MGLL expression and successful SRO in patients with iNOA undergoing micro-TESE. **(A)** Relative expression of circ_MGLL in the testicular tissues of patients with successful and failed SRO. **(B)** Dose–response relationship between the relative expression of circ_MGLL and the probability of successful SRO. The adjustment factors included all baseline covariates: age, BMI, TV, FSH, LH, T, and pathological type. The filled and hollow circles represent the estimated values and their corresponding 95% confidence intervals. circ_MGLL, circular RNA monoglyceride lipase; SRO, sperm retrieval outcome; iNOA, idiopathic non-obstructive azoospermia; micro-TESE, microdissection testicular sperm extraction; BMI, body mass index; TV, testicular volume; FSH, follicle-stimulating hormone; LH, luteinizing hormone; T, testosterone. *P<0.05.

We investigated circ_MGLL levels in paraffin sections of testicular tissues from 114 patients with iNOA. When compared with the level observed in the failure group, the relative expression of circ_MGLL was downregulated in testicular tissues obtained from the successful SRO group (*P* < 0.001, **Table 1**, **Figure 2A**). In addition, a generalized additive model analysis revealed a negative correlation between circ_MGLL expression and the probability of successful SRO (*P* < 0.001, **Figure 2B**). More importantly, although we observed that circ_MGLL expression was higher in patients with SCOS than in those with HS or MA, this difference was not statistically significant (*P* = 0.069, **Table 2**).

**Table 2:** The relationships between circ_MGLL expression and pathological classification of iNOA patients receiving micro-testicular sperm extraction.

Pathological type	SCOS	MA	HS	*P*
**Number**	49	57	8	
c**irc_MGLL**	5.94 ± 2.22	5.34 ± 2.46	3.96 ± 2.01	0.069

SCOS, Sertoli cell-only syndrome; MA, maturation arrest; HS, hypo-spermatogenesis.

### 3.3 Association between circ_MGLL and SRO in patients with iNOA

**Figure 3:**
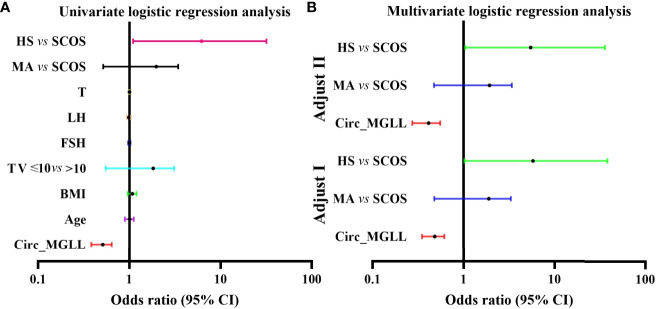
Logistic regression analyses of the clinical characteristics for successful sperm retrieval in patients with iNOA. **(A)** Crude odds ratios in the univariate analyses. **(B)** Adjusted odds ratios in the multivariate analyses. Adjust I, model adjusted for age, BMI, and TV; Adjust II, model adjusted for age, BMI, TV, FSH, LH, and T. circ_MGLL, circular RNA monoglyceride lipase; iNOA, idiopathic non-obstructive azoospermia; BMI, body mass index; TV, testicular volume; FSH, follicle-stimulating hormone; LH, luteinizing hormone; T, testosterone; SCOS, Sertoli cell-only syndrome; MA, maturation arrest; HS, hypo-spermatogenesis.

We conducted univariate and multivariable logistic regression analyses to evaluate the association between clinicopathological features and SRO in patients with NOA. We included circ_MGLL, age, BMI, TV, serum FSH, LH, T, and pathological type in the univariate analysis. As shown in **Table 3**, patients with high circ_MGLL were more likely to experience failed SRO, and patients with the pathological classification of HS were more likely to experience successful SRO in the crude model (all *P* < 0.01, **Table 4**, **Figure 3A**). Other parameters such as age, BMI, TV, FSH, LH, and T were not significantly associated with SRO (*P* > 0.05, **Table 3**, **Figure 3A**). Multivariate analysis was then conducted using circ_MGLL and pathological types based on the significant associations observed in the univariate analysis. After adjusting for baseline covariates, high circ_MGLL expression exerted a significantly negative impact on the probability of successful SRO in patients with iNOA (model 1 adjusted for age, TV, and BMI; odds ratio, OR: 0.470, 95% confidence interval, CI: 0.357–0.620, *P* < 0.001; model 2 adjusted for age, TV, BMI, FSH, LH, T, and pathological type: OR: 0.398, 95% CI: 0.282–0.563, *P* < 0.001, **Table 4**, **Figure 3B**). More importantly, after stratification according to age, BMI, TV, FSH, LH, T, and pathological types, inverse correlations were still observed between circ_MGLL and successful SRO of micro-TESE (all OR <1, **Table 5**).

**Table 3:** Univariate logistic analyses of the clinical characteristics for successful sperm retrieval in idiopathic non-obstructive azoospermia patients.

Variables	Statistics	SR	*P*
c**irc_MGLL**	5.498 ± 2.369	0.500 (0.388, 0.644)	<0.001
**Age (years)**	30.079 ± 3.292	0.998 (0.890, 1.118)	0.967
BMI (kg * m^-2^)	25.105 ± 3.422	1.070 (0.957, 1.196)	0.233
TV (ml)			
**≤10** ml	65 (57.018%)	1.0	
**>10** ml	49 (42.982%)	1.511 (0.711, 3.212)	0.283
Follicle-stimulating hormone **(IU/L)**	21.777 ± 12.246	0.996 (0.966, 1.027)	0.789
Luteinizing hormone **(IU/L)**	9.547 ± 7.527	0.982 (0.929, 1.037)	0.506
Testosterone **(ng/**dl**)**	404.664 ± 185.733	0.999 (0.997, 1.001)	0.570
Pathological type			
**SCOS**	49 (42.982%)	1.0	
**MA**	57 (50.000%)	1.611 (0.728, 3.564)	0.239
**HS**	8 (7.018%)	6.187 (1.121, 34.145)	0.037

BMI, body mass index; TV, testicular volume; SCOS, Sertoli cell-only syndrome; MA, maturation arrest; HS, hypo-spermatogenesis.

**Table 4:** Multivariate logistic analyses of the clinical characteristics for successful sperm retrieval in idiopathic non-obstructive azoospermia patients.

Variables	Adjust I OR (95% CI)	*P*	Adjust II OR (95% CI)	*P*
**circ_MGLL**	0.470 (0.357, 0.620)	<0.001	0.398 (0.282, 0.563)	<0.001
Pathological type				
**SCOS**	1.0		1.0	
**MA**	1.541 (0.688, 3.454)	0.294	1.566 (0.693, 3.542)	0.281
**HS**	6.836 (1.133, 41.259)	0.036	6.223 (1.000, 38.732)	0.050

SCOS, Sertoli cell-only syndrome; MA, maturation arrest; HS, hypo-spermatogenesis; Adjust I, model adjusted for age, volume, and body mass index; Adjust II, model adjusted for age, volume, body mass index, follicle-stimulating hormone, luteinizing hormone, testosterone, and pathological type.

**Table 5:** Stratified analysis of circ_MGLL in testicular tissues for successful sperm retrieval in idiopathic non-obstructive azoospermia patients with different clinical characteristics sub-groups.

Sub-group	*N*	Odds ratio (95% CI)	*P*
Age (years)			
23–28	38	0.401 (0.235, 0.684)	<0.001
29–30	29	0.512 (0.304, 0.862)	0.012
31–41	47	0.531 (0.369, 0.764)	<0.001
BMI (kg * m^-2^)			
17.3–23.5	37	0.456 (0.277, 0.749)	0.002
23.7–26.3	39	0.512 (0.334, 0.784)	0.002
26.5–38.4	38	0.461 (0.282, 0.755)	0.002
TV (ml)			
≤10 ml	65	0.434 (0.292, 0.645)	<0.001
>10 ml	49	0.564 (0.399, 0.798)	0.001
Follicle-stimulating hormone (IU/L)			
2.51–15.58	38	0.506 (0.320, 0.800)	0.004
15.60–24.21	38	0.370 (0.193, 0.709)	0.003
24.24–78.94	38	0.469 (0.307, 0.717)	<0.001
Luteinizing hormone (IU/L)			
1.01–6.47	38	0.597 (0.396, 0.900)	0.014
6.72–10.22	38	0.321 (0.167, 0.617)	<0.001
10.26–62.44	38	0.497 (0.324, 0.764)	0.001
Testosterone (ng/dl)			
156.47–297.64	38	0.529 (0.351, 0.796)	0.002
299.67–425.88	38	0.543 (0.364, 0.811)	0.003
432.90–1,005.41	38	0.354 (0.179, 0.700)	0.003
Pathological type			
SCOS	49	0.565 (0.391, 0.816)	0.002
MA	57	0.418 (0.270, 0.648)	<0.001

BMI, body mass index; TV, testicular volume; SCOS, Sertoli cell only syndrome; MA, maturation arrest; HS, hypo-spermatogenesis.

### 3.4 Construction and validation of circ_MGLL-based nomogram for patients with iNOAs undergoing micro-TESE

**Figure 4:**
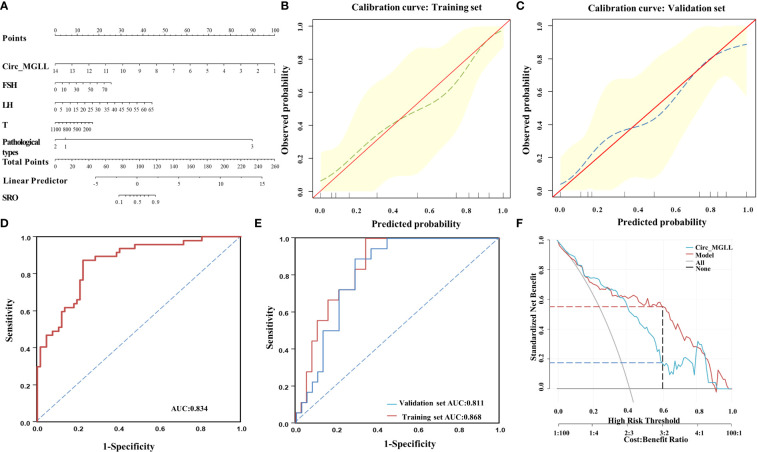
Performance of the circ_MGLL-based nomogram in predicting successful SRO in patients with iNOA. **(A)** Nomogram for successful SRO prediction for the model combining circ_MGLL with clinicopathological variables. **(B)** Calibration curve for the circ_MGLL-based nomogram model in the training set. **(C)** Calibration curve for the circ_MGLL-based nomogram model in the validation set. **(D)** ROC curve for predicting successful SRO based on circ_MGLL in 114 patients with iNOA. **(E)** ROC curve for predicting successful SRO using the circ_MGLL-based nomogram in the training and validation sets. **(F)** Decision curve analysis of the clinical utility of the circ_MGLL-based nomogram for predicting successful SRO for microdissection testicular sperm extraction in patients with iNOA. circ_MGLL, circular RNA monoglyceride lipase; iNOA, idiopathic non-obstructive azoospermia; SRO, sperm retrieval outcome; ROC, receiver operating characteristic; FSH, follicle-stimulating hormone; LH, luteinizing hormone; T, testosterone.

All independent predictors in the multivariate logistic analysis and serum hormone levels that were identified to be associated with spermatogenesis in previous literature were integrated to generate a nomogram for predicting the probability of a successful SRO. Each variable was assigned a value between 0 and 100 according to its contribution to the established model. By summing these values, a total value can be obtained and applied to predict the corresponding probabilities of successful SRO (**Figure 4A**). Using the bootstrap method for internal validation, calibration curves indicated a good agreement between the predicted probability of the circ_MGLL-based nomogram and the observed probability in both the training (**Figure 4B**) and validation sets (**Figure 4C**). Furthermore, in the ROC analysis, the area under the curve (AUC) for circ_MGLL expression to independently predict the SRO of micro-TESE was 0.834, with a cutoff value of 5.354, a sensitivity of 85.1%, and a specificity of 73.1% (**Figure 4D**, **Table 6**). The circ_MGLL-based nomogram demonstrated AUC_t_ values of 0.868 and 0.811 in the training and validation sets (**Figure 4E**, **Table 5**), respectively. These findings indicated that the nomogram exhibited good discrimination and excellent calibration abilities.

**Table 6:** Predictive performance of circ_MGLL-based nomogram.

Variables	Circ_MGLL	Training set	Validation set
**Successful** sperm retrieval outcomes (**SRO**)	47	29	18
**Failure SRO**	67	29	38
**Cutoff value**	5.354	0.268	-0.763
**Sensitivity**	0.851	0.793	0.944
**Specificity**	0.731	0.828	0.658
**PPV**	0.690	0.821	0.567
**NPV**	0.875	0.800	0.962
**Accuracy**	0.781	0.810	0.750
**AUC**	0.834	0.868	0.811
**95% upper limit**	0.755	0.776	0.701
**95% lower limit**	0.899	0.960	0.922
**PLR**	3.168	4.600	2.761
**NLR**	0.204	0.250	0.084
**DOR**	15.556	18.400	32.692

PLR, positive likelihood ratio; NLR, negative likelihood ratio; DOR, diagnostic odds ratios; PPV, positive predictive value; NPV, negative predictive value; AUC, area under ROC curve.

Finally, we performed DCA to determine the clinical potential of the circ_MGLL-based nomogram in assisting with micro-TESE decisions. The gray line (leftmost) represents the micro-TESE strategy for all patients, and the horizontal black line indicates the “micro-TESE for none” strategy. Curves representing the nomograms based on circ_MGLL alone and the integrated factors are shown. As expected, both nomograms were superior to the “micro-TESE for all” strategy (**Figure 4F**). Furthermore, the net benefit of the circ_MGLL-based nomogram was more powerful for patients with threshold probabilities of 40–80% than circ_MGLL alone (**Figure 4F**). These data indicated that the clinical utility of the circ_MGLL-based nomogram was superior to that of a circ_MGLL alone on most occasions.

## 4 Discussion

In the current study, we observed significant differences in circ_MGLL expression between successful and failed SRO among patients with iNOA, whereas other variables (including age, BMI, TV, FSH, LH, and T) did not differ between the successful and failed SRO groups. When compared with the level observed in the failure group, the relative expression of circ_MGLL was significantly downregulated in the successful SRO group. Both univariate and multivariate analyses indicated that circ_MGLL expression was negatively associated with SRO in patients with iNOA, even after stratification according to age, TV, BMI, FSH, T, and pathological type (all OR <1). Notably, our results demonstrated that the circ_MGLL-based nomogram model was a relatively accurate predictor with the clinical potential to assist andrologists in screening patients with iNOA suitable for micro-TESE surgery.

Some molecules in the seminal plasma have been identified as predictors of residual spermatogenesis in patients with NOA (16, 22). However, seminal plasma is a mixture secreted by accessory glands such as the prostate, seminal vesicle gland, and ureteral bulbar gland (23). Thus, the specific expression of molecules in the seminal plasma or seminal exosomes may only partly reflect the spermatogenic function of the testis. Furthermore, although some studies have indicated that spermatogenesis varies in different areas of the testis (24), a series of studies have verified that patterns of expression for testis-specific genes or non-coding RNAs are significantly associated with the success of sperm recovery in patients with NOA, emphasizing their potential value as predictors of successful SRO in these patients (25, 26). In our previous collaboration, we observed that circ_MGLL expression is upregulated in patients with NOA when compared with the level observed in patients with obstructive azoospermia (17). In the current study, we further demonstrated that the expression of circ_MGLL is negatively correlated with the SRO in patients with iNOA, and the circ_MGLL-based nomogram had better diagnostic efficiency in predicting the SRO of micro-TESE for these patients. Further increasing the predictive power of this circ_MGLL-based nomogram by integrating histopathological results and the expression of testis-specific molecular biomarkers may help to avoid unnecessary surgery and excessive financial burden in patients with iNOA.

Previously, we reported that the proliferating speed of Sertoli cells was increased, the proportion of proliferating cells and cells at S stage was upregulated, and the apoptosis levels of Sertoli cells were decreased after silencing circ_MGLL expression (19). Furthermore, bioinformatics analysis and luciferase reporter assays showed that circ_MGLL can bind to miR-1228, miR-1233, miR-149, and miR-924 (19). Previous studies had already demonstrated that miR-1288 expression is abundant in the plasma and testis of cynomolgus monkeys, exhibiting associations with apoptosis and cell differentiation (27). Additional studies have indicated that miR-1233 inhibits apoptosis and promotes the proliferation of A549 cells by targeting dual-specificity phosphatase 9 (28). More importantly, miR-149 expression in sperm is correlated with the quality of early embryonic development (29). Previous literatures proved that Sertoli cells provide physical support and stable microenvironments or niche for developing sperm and perform an important role in sustaining spermatogenesis (30, 31). Considering the important functions of circ_MGLL and its targeted miRNAs in cell apoptosis, we speculate that a regulatory network with circ_MGLL as the core would induce apoptosis and the dysfunctions of Sertoli cells in patients with NOA, which may further result in the residual sperm losing nutritional supports and relatively normal microenvironments. Consequently, this dysfunction of Sertoli cells induced by circ_MGLL upregulation will eventually lead to the failure of micro-TESE. However, the mechanism by which circ_MGLL regulates cell proliferation and the functions of Sertoli cells and spermatogenesis should be further explored in future studies.

Notably, our results also indicated that the serum levels of FSH, LH, and T were not correlated with the outcome of sperm collection and could not be used as reliable predictors of SRO in patients with iNOA undergoing micro-TESE. However, findings regarding the correlation between hormonal levels and SRO have been inconsistent. Consistent with our results, some preliminary studies of patients with NOA have reported no significant differences in TV or hormonal levels based on the success or failure of micro-TESE (32, 33). Accordingly, serum hormone levels were not identified as a significant predictor of successful SRO (34). However, some studies have reported significantly higher levels of serum FSH in the failed group than in the successful group. This may be because the abovementioned studies failed to exclude patients with Klinefelter syndrome (KS) and AZF c microdeletions. Patients in these latter two groups may exhibit less remarkable increases in FSH as well as a relatively higher success rate following sperm collection than those with iNOA (5, 35, 36). In contrast, our study excluded patients with KS and AZFc microdeletion, instead focusing on patients with iNOA, which is often associated with significantly elevated FSH levels and low sperm retrieval rates (36, 37). Despite the associations observed in the current study, further research is required to clarify the effects of hormonal levels on SRO in patients with iNOA as well as the mechanisms underlying their actions.

In addition to the retrospective nature of this study, some non-negligible limitations exist—for example, all testicular tissues used for the detection of circ_MGLL were paraffin-embedded testicular tissues obtained during micro-TESE surgery, and we did not examine the testicular biopsy tissue obtained *via* needle aspiration prior to micro-TESE surgery. Thus, for patients who meet the criteria of testicular needle aspiration, such an analysis may help to verify our findings. Furthermore, although we observed that the circ_MGLL-based nomogram could reflect the focal spermatogenesis of the whole testis and could be used as a predictor for SRO in patients with iNOA, the expression profiles and the function of circ_MGLL in germ cells and testicular tissues should be explored in future studies.

In summary, the detailed data obtained in our study demonstrate that higher circ_MGLL levels are associated with decreased success in sperm retrieval *via* micro-TESE in patients with iNOA. More importantly, to the best of our knowledge, the present study is the first to establish a competing risk nomogram for calculating the probability of successful SRO, which may aid in screening for micro-TESE eligibility. Although these results provide evidence regarding the significant association between circ_MGLL expression and failure of micro-TESE in patients with iNOA, an additional large-sample multicenter prospective cohort study is required to further validate the efficacy of the circ_MGLL-based nomogram for predicting successful SRO.

## Data availability statement

The original contributions presented in the study are included in the article/**Supplementary Material**, further inquiries can be directed to the corresponding author.

## Ethics statement

The studies involving human participants were reviewed and approved by the Ethics Review Board of the Northwest Women’s and Children’s Hospital (ethical review number: 2021-XBFE-023). The patients/participants provided their written informed consent to participate in this study.

## Author contributions

MW designed the research study. SS, TW, and LW performed the research. SS analyzed the data. MW and SS wrote the paper. All authors contributed to the article and approved the submitted version.
