# Supplementary figures and images for: Nomogram based on a circular RNA biomarker for predicting the likelihood of successful sperm retrieval via microdissection testicular sperm extraction in patients with idiopathic non-obstructive azoospermia
Source: Front Endocrinol (Lausanne). 2023 Jan 17;13:1109807. doi: 10.3389/fendo.2022.1109807 (PMC9886672; doi:10.3389/fendo.2022.1109807)

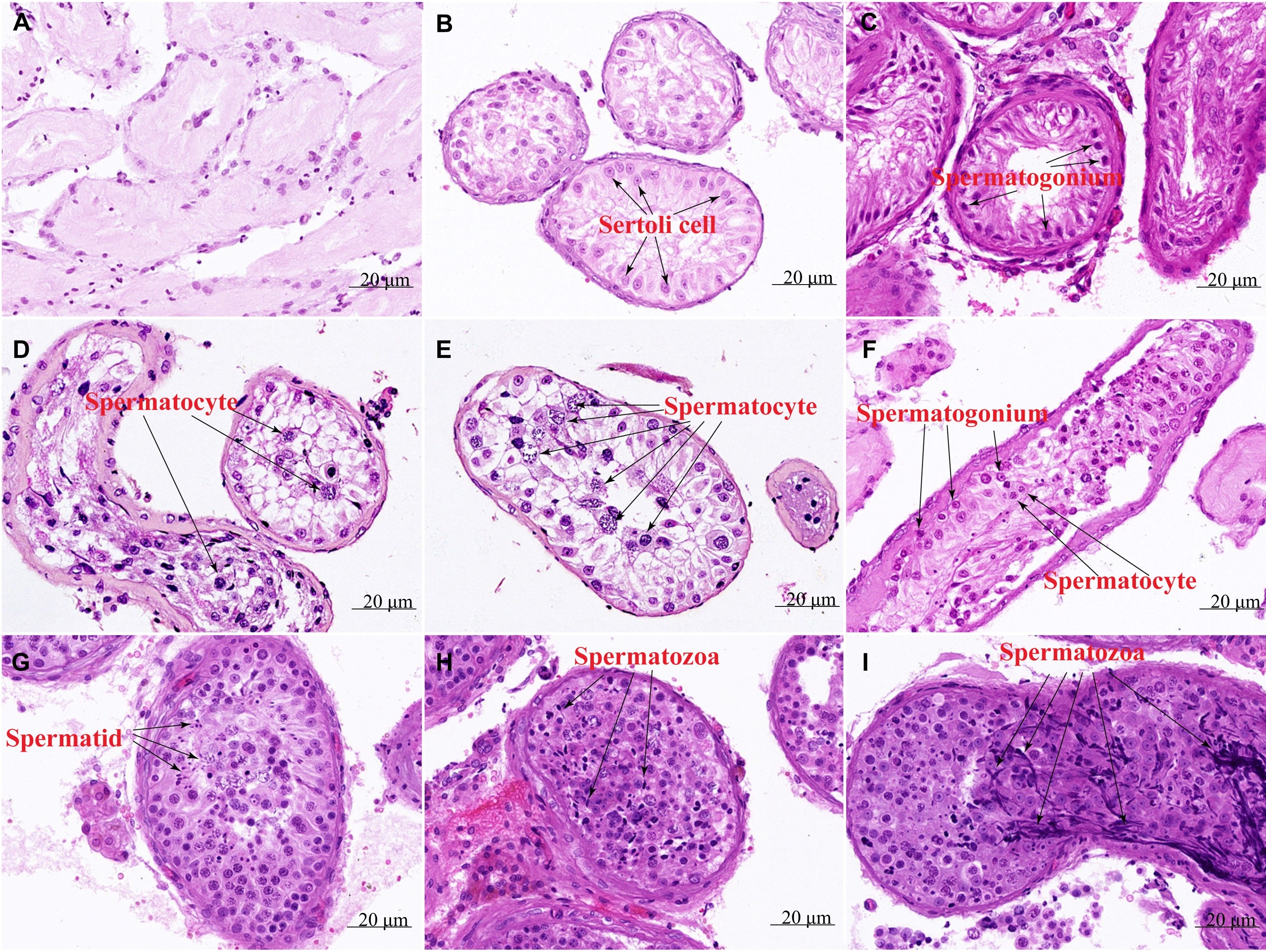

Supplement: Supplementary file 1 [file Image_1.jpeg]
